# Comparative Assessment of Hypersensitivity Reactions on Use of Latex and Nitrile Gloves Among General Dental Practitioners: A Cross-Sectional Study

**DOI:** 10.7759/cureus.46443

**Published:** 2023-10-03

**Authors:** Naleen Naranje, Priyanka Paul, Kshitija P Parate, Amit Reche

**Affiliations:** 1 Public Health Dentistry, Sharad Pawar Dental College and Hospital, Datta Meghe Institute of Higher Education and Research, Deemed to be University, Wardha, IND

**Keywords:** swelling, rashes, erythema, itching, dentist, allergy, hypersensitivity, nitrile, gloves, latex

## Abstract

Background

Latex gloves are used more frequently by dental, medical, and other health workers and their allergy has also increased as a result, dentists are frequently exposed to latex or nitrile gloves for extended periods. This prolonged exposure often leads to local symptoms such as itching and erythema rashes. However, some dentists experience more severe systemic reactions, including swelling, wheezing, breathlessness, and even an increase in blood pressure. Latex gloves have recently been replaced with nitrile gloves, powder-free latex gloves, and other preventive measures to avoid allergies. Latex allergies are more common than nitrile allergies, as they are hypoallergic with properties such as tear-resistant and provide an equivalent level of defense against various dental materials and procedures. Women experience more allergic reactions than men. Not only are dentists exposed to this allergy but the patient can also be exposed during the procedure. This study aimed to assess hypersensitive reactions to the use of latex and nitrile gloves among general dentist practitioners and dental students.

Methods

A cross-sectional study was conducted in Wardha, Maharashtra, India, to evaluate allergic reactions to latex and nitrile gloves among general dental practitioners and dental students at college. A questionnaire-based study was conducted with a sample size of 356. The self-administered survey inquiries about glove compliance, the time they wear the gloves, regular glove use, and problems related to latex or nitrile contact. In addition, dentists and dental students' personal histories of allergies to medications, dental materials, disinfectants, or other chemicals were noted, as well as signs and symptoms they experienced from prolonged contact with gloves.

Result

The total number of responses collected was 356. The investigated dentists were 274 and 82 were students, out of which 122 (34.3%) were male and 234 (65.7%) were female. Responses showed that 224 (62.92%) used latex gloves, and 132 (37.08%) used nitrile gloves. Among 356 participants 175 showed symptoms by the use of both latex and nitrile gloves, out of which 85.14% showed allergy to latex and 14.85% to nitrile gloves. All 175 individuals showed type IV hypersensitivity, and none of them showed type I.

Conclusion

Latex gloves are not the only option for dentists who experience itching when wearing gloves; they can also use powered-free latex gloves and nitrile gloves or take precautions such as not using oil-based cream, washing their hands, or taking pharmaceuticals such as cetirizine, pheniramine maleate, etc. However, when symptoms worsen and include erythema, swelling, wheezing, and in some cases, anaphylactic shock may occur they tend to use alternative gloves.

## Introduction

The usage of latex dates back to 1600 B.C. but the first precise description was not made public until 1979 which showed that a patient with a history of persistent atopic dermatitis presented with hand pruritus five minutes after donning rubber gloves, she displayed an instantaneous wheal with a rubber glove extract when tested using the skin prick method. Natural rubber latex (NRL) comes from the tropical Hevea Brasiliensis tree which is used to make gloves and other dental materials. Disposable NRL gloves are a crucial component of infection prevention in dental and routine procedures. Using low-allergen, powder-free latex gloves has dramatically decreased the incidence of latex allergy [1]. Rash, type I allergic reaction, and allergic contact dermatitis (delayed-type hypersensitivity reactions) are some serious complications associated with latex gloves. Exposure can occur through powder contact through the respiratory tract, the membrane of the nose. Diasdeandrade et al. show that in patients undergoing treatment such as root canal treatment, extraction, and other dental procedures that allow contact with latex gloves and material such as rubber dam shows that after one to two minutes of contact, some patients who are allergic to latex show anxiousness with erythema in the contact area, which shows swollen lips, rise in local temperature, itching, increased blood pressure, dyspnea, and exophthalmos. After observing these signs and symptoms, oxygen and 100 mg hydrocortisone were injected immediately, and the patient was kept under observation. This can happen to health workers who do not know that they are sensitive to latex [2]. Latex emerged as a substantial occupational health threat in the mid-1980s and the pandemic. In countries such as the US and UK, nitrile gloves are generally used more than latex gloves, whereas, in other countries, latex gloves are more commonly used because they are easily accessible and economical [3].

Some dentists who are sensitive to latex and experience rashes, and erythema avoid contact with latex and replace it with nitrile or vinyl powder-free gloves and some take precautions like applying topical and oral steroids which help lessen symptoms. During pandemics, such as the recent COVID-19 epidemic, nitrile and latex gloves are preferred. Latex gloves are adaptable, comfortable, and tactilely sensitive and offer a medium degree of protection, whereas vinyl gloves offer mediocre protection and are tactilely responsive but are less sturdy [4]. It was also found that this allergy caused by type I hypersensitivity may occur within 20-30 min of exposure and may include itching, flushing, swelling in the area of contact, difficulty in breathing, increased heart rate, and anaphylactic shock. Along with the skin test, a radioallergosorbent (RAST) test is performed for patient and dentist safety EpiPen and Benadryl Elixir are informed to carry to prevent allergic symptoms caused by anaphylactic shock. The most commonly used glove in India is latex because it is cheap and easily available. With the increase in allergy and pro-quality of nitrile gloves are used by many dentists and studies because they are made of a distinctive sort of polymer that provides superior chemical and tear resistance at the same time, they do not use powder so allergies from powder are also prevented, they are stronger and tear resistance is also high [5].

Other dental materials that contain rubber latex include gloves, rubber dams, cartridges, tourniquets, rubber bands, and resuscitation equipment are also made of latex not just dental practitioners, but health workers and patients can also be affected by this allergy. A complete history of allergic responses should be obtained from potential patients. Nitrile is better than latex because it is thinner than latex and still does not tear off, and its frequency of allergic reaction is much lower than that of latex [6]. A type of symptomatic dermographism known as glove-related hand urticaria is believed to be brought on by a mix of shearing forces from repeated donning and removing of gloves and direct pressure from the glove. Healthcare professionals are more prone to develop glove-related hand urticaria because they use gloves frequently, and based on recent patterns in our practice, we believe that this is an occupational issue that is worsening [7]. Despite improvements following the adoption of powder-free NRL gloves, a sizable proportion of hospital workers continue to experience discomfort associated with NRL. Therefore, an additional study on latex avoidance and the reason for lingering allergic signs in workers with NRL allergy is required. Rather than gloves, products containing latex, such as protection, also show allergic reactions that are often confused with infection and are most commonly seen with contact dermatitis, contact urticaria, and, more rarely, anaphylaxis [8].

Glove allergy care requires identifying the source of the immunologic reaction, followed by confirmation of the diagnosis if type I lgE-mediated allergy is expected. People with gloves allergies are frequently treated with corticosteroids and antihistamines to alleviate nasal, ocular, cutaneous, and respiratory issues. After using latex gloves, all dentists and healthcare workers who are suffering from allergies should wash their hands thoroughly. The Lowry test, also known as the latex enzyme-linked immunosorbent assay for antigenic protein (LEAP), is used to quantify latex protein, which should be less than 10 mg/g to exhibit the least allergenicity [9]. Vinyl gloves are not recommended for general safety measures by the National Institute for Occupational Safety and Health because they do not provide an effective barrier against HIV [9].

Numerous studies have demonstrated that latex gloves are allergic in the health care profession however there are only a few studies about nitrile glove allergy. In our study, we have compared both latex and nitrile allergy and which gloves are more allergic to our fellows and dental practitioner through our study we have highlighted their symptoms after wearing gloves and which glove is more allergic to the dental practitioner, also research help to educate their level of understanding on how to prevent this allergy and what is the alternative to prevent allergy. Although gloves are something every practitioner wears, allergies caused by them are not well acknowledged. Therefore, the purpose of this research was to identify which glove is hyperallergic among dental students and practitioners.

## Materials and methods

A cross-sectional study was conducted in Wardha, Maharashtra, India, to evaluate the allergic reactions to latex gloves and nitrile among dentists and dental students who are practicing clinical work and studying at Sharad Pawar Dental College and Hospital a questionnaire-based study was initiated after obtaining approval from the Institutional Ethical Committee (DMIHER(DU)/IEC/2023/722), Sharad Pawar Dental College and Hospital DMIHER. Details were received from dentists aged 18-60 years which were self-explanatory working at Sharad Pawar Dental College, Sawangi, Wardha, which is situated in the central part of India. The study was conducted for four months, from April 2023 to July 2023. The study sample consisted of 365 subjects. The study was specific to the dental profession only, so we included only dental students and practitioners and excluded other health workers.

Google Forms (Google, Inc., Mountain View, CA, USA) was used for recording the responses of the study and dentist participants. Questionnaires were sent to the participants through e-mail and WhatsApp, and responses were recorded. Statistical analysis was done using the Statistical Package for the Social Sciences (SPSS) version 21 (IBM Corp., Armonk, NY, USA) and Microsoft Office Excel (Microsoft Corp., Redmond, WA, USA). The chi-square test was used to compare categorical variables. Statistical significance was determined at P-values of less than 0.05 using the chi-square test used to compare categorical variables. The self-management questionnaire included 25 questions regarding conformance, which included both closed-ended questions and Open-ended questions. The question was formulated in two sections; The first section includes demographic details, which mainly comprise participant age, gender, years of practice, and qualification. The second section included both open-ended and close-ended questions, out of which nine questions were “yes” and “no” type and 11 were open-ended to know the various symptoms dentists experience during work. Questions based on, types of gloves, regular glove use, and symptoms related to latex and nitrile contact were included in the online survey. In addition, dentists' own experiences of allergies to drugs, dental materials, disinfectants, or others were noted, as well as allergies to vegetables, fruits, or others. And what precautions they use to prevent it (Table 1).

**Table 1:** Questionnaire for the assessment of latex and nitrile allergy.

Questions	Options
Are you a regular dental practitioner?	Yes
	No
Which type of gloves do you use regularly?	Latex
	Nitrile
	Other
Any family history of allergy?	Yes
	No
Do you have any systemic condition?	
How many hours do you work a day?	4-5hr
	6-7hr
	8-9hr
	10-12hr
How many hours do you wear gloves during dental practice?	1-2hr
	2-3hr
	4-5hr
	5-6hr
	8-9hr
Do you have any history of rashes on your hand after the use of gloves?	Yes
	No
Do you have a history of anaphylaxis?	Yes
	No
Do you have any other symptoms?	Itching
	Erythema
	Swelling
	Wheezing
	None of the above
When you wear gloves have you noticed any?	Shortness of breath
	Chest tightness
	Other
	None of the above
Have you undergone any allergy skin test?	Yes
	No
Do you suffer from any other allergy?	Yes
	No
If yes which kind of allergy?	Dental materials
	Drug
	Disinfectant
	Soap
	Others
Do you suffer from food allergy?	Vegetable
	Fruits
	Other
Do you have any history of water vesicles on your hands or crusted skin?	Yes
	No
Have you experienced swelling or difficulty in breathing after blowing off a balloon?	Yes
	No
Do you have any history of frequent surgery or invasive medical procedure?	Yes
	No
How do you manage the glove allergy?	Quit wearing gloves
	General medical treatment
	Other
What precaution do you take to prevent glove allergy?	Avoiding oil-based cream
	Handwash after wearing gloves
	Medication
	Use alternative gloves
Do prolong use of rubber dam during procedure cause any changes like?	Swollen lips
	Itching in mouth and tongue
	Ulcer
	All of the above
	None of the above

## Results

**Figure 1:**
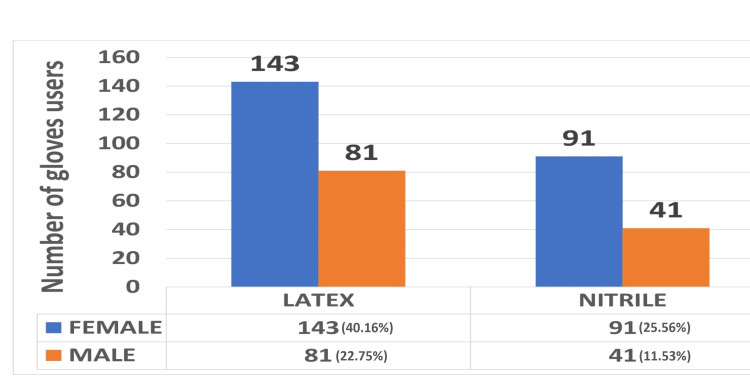
Graphical representation of subjects using latex and nitrile gloves.

The total number of responses collected was 356. In which investigated dentists were 274 and students 82 out of which 122 (34.3%) were male and 234 (65.7%) were female. Of the 356 responses, 224 (62.92%) used latex gloves, 132 (37.08%) used nitrile gloves, and none of them used other gloves as shown in Figure 1.

**Figure 2:**
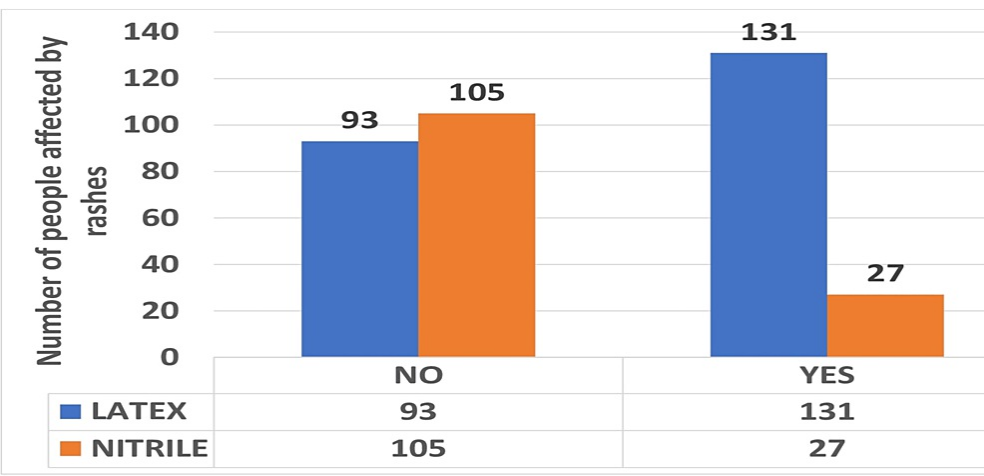
Graphical representation of the subjects exposed to rashes due to latex and nitrile gloves.

The number of years of clinical activity ranged from one to 35 years; 209 (58.70%) had under 1-10 years of professional activity, 73 (20.50%) had between 11 and 20 years, 63 participants had 21-30 years of experience, and 11 (3.08%) had more than 31 years and above. In our study, 62.92% of dentists and students used latex gloves for prevention, and 37.07% used nitrile gloves. Since the implementation of the Universal Precautions concept in 1987, sensitivity to latex gloves has become a significant professional concern among healthcare workers, dental staff, and dental students, especially for those who frequently wear these gloves. In our study, 44.38% of the investigated dentists and students reported signs of rashes because of the use of gloves and 55.61% did not experience any rashes due to gloves. Of 158, 131 (82.91%) were latex gloves users and 27 (17.09%) were nitrile users (Figure 2).

**Figure 3:**
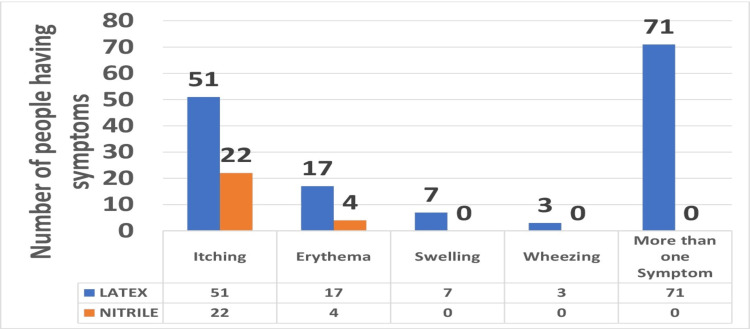
Comparative Graphical representation of symptoms between latex and nitrile gloves.

Out of 356 responses, 175 (49.15%) reported symptoms affecting the hand skin in straight contact with the latex and nitrile comprising: erythema 21 (12%), itching 73 (41.71%), and swelling seven (2.28%), suggesting allergic dermatitis. Other signs include systemic responses such as allergic rhinitis, allergic conjunctivitis, cough, and wheezing three (1.71%). None of them described severe reactions to gloves, such as anaphylactic shock. Local symptoms for latex gloves users were remarkably more common among female dentists than male dentists. Females show symptoms such as itching 36 (70.58%), erythema 13 (76.47%), and swelling five (71.42%) while the percentage for males are itching 15 (29.42%), erythema four (23.53%), and swelling two (28.58%). Out of 356 responses, 175 participants showed symptoms out of which 149 (85.51%) were latex users: itching 51 (69.86%), erythema 17 (80.88%), swelling seven (100%), and wheezing three (100%) and nitrile users were 26 (14.85%): itching 22 (30.13%), erythema four (19.04%), swelling nil, and wheezing nil (Figure 3, Table 2).

**Table 2:** Symptoms between latex and nitrile gloves.

SYMPTOMS	LATEX (149)				NITRILE (26)			
	FEMALE (55.43%)		MALE (29.72%)		FEMALE (8.57%)		MALE (6.28%)	
	N	%	N	%	N	%	N	%
ITCHING	36	70.58%	15	29.41%	14	63.63%	8	36.36%
ERYTHEMA	13	76.47%	4	23.52%	1	25%	3	75%
SWELLING	5	71.42%	2	28.57%	nil	00%	nil	00%
WHEEZING	3	100%	nil	00%	Nil	00%	nil	00%
MORE THAN ONE SYMPTOMS	40	56.33%	31	43.66%	nil	00%	nil	00%

**Figure 4:**
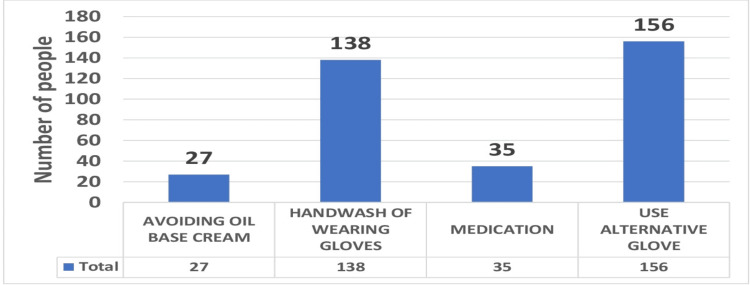
Graphical representation of precautions taken to prevent glove allergy among the total subjects.

The results showed that the age group of 35-50 years was most frequently affected by latex glove allergic reactions. Of 203 dentists whose professional experience is 5-30 years, 121 (59.60%). The younger demographic has not yet been often subjected to latex, and older practitioners were not accustomed to using protective gloves at the beginning of their professional activity, which may be one factor. The dentists’ medical history confirmed the prevalence of additional allergic reactions, including food allergies from fruits at 3.37%, vegetables at 5.05%, allergies to different drugs at 8.70%, and allergic asthma at 8.4%. It has been shown that those who are allergic to particular meals also suffer from an allergy to latex, whereas participants who are allergic to nitrile were nil. Latex and nitrile allergies have also been linked to sensitivity to dental materials by 3.65%, disinfectants by 4.77%, and soap by 2.52%. Most practitioners wore sanitized gloves for more than 1-2 h per day (7.86%), 2-3 h per day (22.7%), 4-5 h per day 18.25%, 5-6 h per day 22.19%, and 8-9 h per day 28.93%. With a substantially higher percentage of female practitioners than male dentists, female dentists appeared more involved with hand protection. Over 182 (51.12%) wore latex gloves for more than five hours a day out of which 128 were latex users and 54 were nitrile users, of which symptomatic percentage were symptomatic. Findings regarding the association between regular glove use hours and the rate of latex glove allergy among dentists wearing gloves for more than five hours per day than those wearing gloves with nitrile. Only 39 (10.95%) dentists in our study underwent a skin test aimed at identifying the individual risk and specific allergens. Skin testing provides the possibility of unbiased determination of latex or nitrile. The latex-sensitive dentists' approach to allergic reactions consisted of preventing contact with natural latex by using non-latex (e.g., nitrile or vinyl) powder-free gloves (92.9%) instead of natural latex gloves. Of the participants, 1.12% experienced water blisters, three experienced it due to latex gloves, and only one participant experienced it from nitrile. The most common precautions taken to prevent allergy by dentists and dental students were avoiding oil base cream 7.58%, hand washing 38.76%, medication 9.83%, and use of alternative gloves 43.82% (Figure 4).

## Discussion

Disposable NRL gloves are a crucial component of infection prevention in dental and routine procedures and allergy to latex gloves is a major occupational problem [10]. It is required to wear gloves during dental procedures because they can significantly reduce the amount of blood that is transferred from a needle stick incident as well as protect us from different chemicals and materials, along with drugs, from direct contact with our skin. In our study both latex and nitrile gloves were included with a sample size of 356 showing a response of 175 participants showing symptoms, out of which latex users were 149 (85.51%) with symptoms like itching 51 (69.86%), erythema 17 (80.88%), swelling seven (100%), and wheezing three (100%), and nitrile users were 26 (14.85%): Itching 22 (30.13%), erythema (19.04%), swelling nil, and wheezing nil. With the use of gloves, the cases of hypersensitivity increased, research conducted by Barlean et al. which was specific to latex glove allergy showed that skin in direct contact with the latex experienced discomfort and other symptoms such as redness (74.5%), itching (42.4%), and swelling (20.1%), suggesting allergic dermatitis which clearly shows that dental healthcare professionals in Iasi, were experiencing symptoms due to latex allergy, three different reactions to latex gloves have been recorded, such as symptoms of irritation, which is typically reversible; delayed hypersensitivity, which is more frequent when exposed to latex proteins and their content; and sensitivity to natural protein residue present in natural latex, which causes immediate hypersensitivity (Type I). This is also known as an immunoglobulin E (IgE) reaction, and it often manifests effects within 5-30 minutes of contact with latex [10]. 

Multiple research indicated that both latex and nitrile sensitivity have been noticed, but more cases of latex sensitivity have been noticed along with this type 1 hypersensitive reaction, which was seen with the use of a rubber dam and confirmed by the presence of IgE antibodies against latex. Rubber dam is the most commonly used material in dental procedures keeping that in mind our survey result shows that 17.13% of participants showed symptoms such as swelling, ulcers, and swollen lips after the use of a rubber dam. This shows that not only the dentist but also the patient we are dealing with are exposed, a study performed by Kleier et al. showed that after the application of a rubber dam, the patient was tense and aroused one minute after applying the dam, and there was obvious erythema on the face, neck, upper body, and upper limbs, as well as facial edema, a rapid heartbeat, and breathlessness [11]. The gold standard for diagnosis is skin-prick testing in patients with localized symptoms and latex-specific IgE antibody assessment in patients with systemic symptoms. Powdered gloves increase the possibility of allergy, not only for the person wearing them but also for any other worker in the same space [12]. Multiple studies also showed that the severity of NRL-related symptoms dropped from 8.5 before to 2.3 after using powder-free NRL gloves were put into place, according to a scale of 0 to 10 [13].

A number of studies showed allergic reactions to latex gloves, but it is also prudent here to mention that in study conducted by Gonzalo-Garijo et al. showed that acute reactions (contact urticaria, rhinoconjunctivitis, or both) were seen in their hospital's staff members who were earlier diagnosed with IgE-mediated hypersensitivity to latex and shown allergic to nitrile, but after using three lots of nitrile gloves, they were able to endure nitrile gloves from different lots made by similar or different manufacturers, and their count was also less than latex [14]. In our study, there were 356 participants, with 85.51% allergic to latex gloves and 14.85% allergic to nitrile this indicate that allergy can occur from both glove but more frequently by latex [14]. Basic therapy for this type of hypersensitivity includes topical corticosteroids, antihistamines, and, in severe cases, systemic corticosteroids. It is also noted that people who have an allergy to these gloves are also allergic to different products such as dental materials, disinfectants, and drugs, including penicillin, sulfonamides, cephalosporins, and anesthesia (local and general) [15]. Whenever a patient shows a history of allergies, nurses should ensure that every operation, whether small or large, is latex-free during the whole perioperative period, ideally moving the surgery to the first hour to ensure that the level of aeroallergens is as low as feasible [16]. According to the experience of dentists and dental students, the use of powder-free, low-protein latex gloves dramatically lowers the incidence of latex allergy and latex-induced asthma compared to powdered latex gloves [17].

Our data also emphasize that patients undergoing multiple surgical procedures and exposed to gloves multiple times were severely affected by latex allergy out of 356, 18 (5.05%) participants underwent frequent surgery and were allergic to it, it is also shown in a study conducted by Yeh et al. on meningomyelocele patients undergoing multiple surgical procedures, the study was conducted in 11 patients, of whom seven were allergic to latex and four were asymptomatic; hence, this study shows that patients with a disease like meningomyelocele who undergo multiple procedures not only the medical practitioner but the patient also suffer from latex allergy [18].

It is noticed that not just doctors, but nurses, patients, dental hygienists, technicians, and other health workers also suffer from allergies. As allergic reactions to latex and nitrile gloves have been identified, manufacturers have responded by offering a broader range of glove types, e.g., vinyl gloves. Despite the fact that vinyl gloves are inferior to latex gloves, some workers prefer them since they are powder-free and do not irritate the skin as easily as powdered latex gloves [19].

Limitations

This study only provides a snapshot of hypersensitivity reactions among dental professionals and may not be generalizable to other populations or settings. The study has limitations related to its scope, gender distribution, and the absence of longitudinal data.

## Conclusions

This study compared the use of latex and nitrile gloves in India. Latex gloves are widely adopted in Indian hospitals due to cost-effectiveness and availability. However, latex allergies are prevalent, with symptoms subsiding when using powder-free latex or nitrile gloves. Additional precautions include avoiding oil-based creams, handwashing, and medication. Asthmatic individuals and those with over five years of work experience exhibit higher allergy rates. Western countries prefer nitrile gloves due to lower allergy rates. Females, long-hour practitioners, and those with allergies to certain materials and foods show increased glove allergy risk. Dentists in private clinics prefer nitrile gloves for their superior quality, hypoallergenic properties, and better dexterity.
